# Development and Evaluation of Chickpea and Flaxseed‐Based Porridge as a Dietary Intervention for Hyperlipidemia in Rats

**DOI:** 10.1002/fsn3.71200

**Published:** 2025-11-25

**Authors:** Sadia Zahid, Uswa Khawar, Rehma Gulzar, Usman Haider, Ghalia Shamlan, Isam A. Mohamed Ahmed, Cristina Maria Maerescu, Florin Leontin Criste, Claudia Terezia Socol, Rana Muhammad Aadil

**Affiliations:** ^1^ Department of National Institute of Food Science and Technology University of Agriculture Faisalabad Faisalabad Punjab Pakistan; ^2^ Department of Institute of Physiology and Pharmacology University of Agriculture Faisalabad Faisalabad Punjab Pakistan; ^3^ Department Food Science and Nutrition College of Food and Agricultural Sciences, King Saud University Riyadh Saudi Arabia; ^4^ Department of Genetics, Department of Animal Science and Technology University of Oradea Oradea Romania

**Keywords:** chickpea, flaxseed, functional foods, high‐fat diet (HFD), histopathology, hyperlipidemia, lipid profile, porridge, synergistic effect

## Abstract

Hyperlipidemia is a condition of elevated blood lipids. Many natural foods possess hypolipidemic properties when consumed regularly. Purposely in this research chickpea and flaxseed‐based porridge was evaluated for its lipid‐lowering effect. A 45‐day therapeutic trial was conducted using 20 male Wistar rats, which were divided into five groups having four rats in each group. For the induction of hyperlipidemia for all rats, a 45% high‐fat diet (HFD) in the form of Vanaspati ghee was mixed with a 55% control diet and was given for 22 days. The experiment included five groups: G0 (normal control), G1 (hyperlipidemic control), and G2–G4 (hyperlipidemic rats) treated with formulated porridge blends T1 (2:1), T2 (1:2), and T3 (1:1) of chickpea and flaxseed for 23 days. Each rat received 30 g of feed daily, comprising 12 g porridge and 18 g basal diet. Lipid profile, liver function tests (LFTs), and renal function tests (RFTs) were evaluated after the intervention. The lipid profile revealed a significant improvement. Liver biomarkers also showed marked reduction. RFT results indicated a significant decrease in urea levels, while creatinine showed nonsignificant changes. Histopathological analysis of the liver, kidney, and heart showed improved tissue structures, with T3 exhibiting the greatest overall improvement due to the balanced ratio of chickpea and flaxseed.

## Graphical abstract

Chickpea‐flaxseed porridge effectively improved lipid profile. Natural foods contain medicinal property exert hypolipidemic effect. Balanced T3 formulation pronounced most significant improvement. Liver and renal biomarkers improved, protective effects on vital organs.

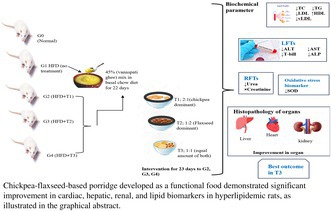

## 1 Introduction

Hyperlipidemia, common in wealthy nations, is the leading cause of coronary heart disease (CHD) due to excess blood fat. Although these fats are necessary for bodily functions, high levels of them can lead to atherosclerosis, which greatly raises the risk of CHD (Mainieri et al. 2023; Zeeshan et al. 2023). Plants have traditionally been a vital source of both nutrition and bioactive compounds, continuing to be a valuable gift from nature that promotes overall health. Since ancient times, natural foods and their derivatives have been acknowledged and utilized as a primary source of medicinal remedies (Anwar et al. 2022; Waseem et al. 2021). Such medicinal plants have been employed since antiquity to manage and cure various health conditions, offering significant therapeutic benefits with few side effects. The rising demand for polyunsaturated fatty acids (PUFAs) as functional food components has led to efforts to enrich traditional diets, aiming to boost nutritional value, support overall health, and aid in disease prevention (Konieczka et al. 2017; Manzoor et al. 2022; Haryati et al. 2025). Controlled trial evidence supports the intake of dietary pulses like peas, lentils, beans, and chickpeas as an effective strategy for improving dyslipidemia. A well‐balanced diet serves as the first‐line treatment for hyperlipidemia (Farzana et al. 2017). Among legumes, chickpeas though an ancient crop have gained increased attention in recent years due to their status as a complete protein, absence of common allergens, and lack of phytoestrogens. Chickpea powder has been incorporated into infant foods and used in protein isolates containing over 70% protein (Bidura 2024). A healthy diet for the elderly not only meets their macro‐ and micronutrient needs but also plays a crucial role in preventing and managing age‐related conditions, including noncommunicable diseases (Mohamed et al. 2020).

Chickpeas have more dietary fiber as compared to grains and oil seed beans. The impact on blood lipid metabolism of chickpea flour and its constituents, including plant sterols, triterpenoid saponins, isoflavones, and α‐galacto‐oligosaccharides (α‐GOS) (Han et al. 2021). Regular consumption of flaxseeds ( *Linum usitatissimum* L.) is an effective strategy to meet the nutritional needs for alpha‐linolenic acid (ALA), as flaxseeds are among the richest natural dietary sources of ALA (Rabail et al. 2023). Flaxseeds are an excellent source of soluble fiber, insoluble fiber, omega‐3 fatty acids, and lignans (secoisolariciresinol di‐glucoside [SDG]) (Esmail et al. 2022). Soluble fibers consist of acidic polysaccharides and neutral glucose chains, whereas insoluble fibers are primarily composed of cellulose, lignin, and acid detergent components (Imran et al. 2024). Flaxseed administration significantly reduced TC, TG, and LDL‐C. Nevertheless, there were no notable impacts on HDL‐C. As previously stated, the lignan, phenolic, and ALA content of flaxseed is what causes these positive dyslipidemia effects (Hamza and Almansour 2024). Because of its high fiber content, flaxseed helps prevent dyslipidemia by increasing satiety, reducing calorie intake, and shortening the time between meals. Through increased fecal excretion of cholesterol, it also decreases bile acid reabsorption and promotes bile acid excretion (Buckner et al. 2016; Harini et al. 2015). Porridge is, a modern food and is, widely consumed across all age groups from children to the elderly (Putra et al. 2021). In this study, the antihyperlipidemic effects of chickpea and flaxseed were investigated in rats. For the administration of these both medicinal plant sources, porridge was formulated.

## 2 Materials and Methods

### 2.1 Procurements of Raw Materials

The required raw materials including chickpea, flaxseeds, carrots, and cinnamon powder were purchased from the local market of Faisalabad, Pakistan. The chemicals needed for the research analysis were obtained from a scientific store.

### 2.2 Preparation of Chickpea and Flaxseed Porridge

Chickpeas and flaxseeds were roasted on low flame and ground into granules and fine powder, respectively. Carrots were cleaned, dehydrated for 3–4 days, and blended into chunks, while cinnamon was ground into powder. A pan was taken, and 2 cups of water with measured porridge powder were added. It was cooked for 20–25 min over medium heat until it reached a porridge‐like consistency. Then, the heat was turned off.

### 2.3 Proximate Analysis of Porridge

The proximate composition of chickpea and flaxseed‐based porridge was determined following the methods described by AACC (2016). Moisture content was analyzed using the hot air oven method, crude fat by Soxhlet extraction, and crude protein by the Kjeldahl method (digestion, distillation, and titration). Ash content was determined using a muffle furnace (MF1/02, PCSIR, Pakistan), while crude fiber was analyzed with the Labconco Fibertech system (Labconco Corporation, Kansas, USA).

### 2.4 Experimental Design

A 45‐day therapeutic trial was conducted to evaluate the effect of chickpea and flaxseeds‐based porridge as a dietary intervention for hyperlipidemia in rats. For this purpose, 20 rats were kept in the animal room at the University of Agriculture, Faisalabad, under the National Institute of Food Science and Technology, with the consent of the Institutional Biosafety and Bioethics Committee (D#2275/ORIC) for 45 days for the efficacy trial. The rats were categorized into five groups (G0, G1, G2, G3, G4), four rats in each group. G0 was the negative control group, G1 was the positive control group, and the other three groups, G2, G3, and G4 were the porridge groups 2, 3, and 4. For the induction of hyperlipidemia, a 45% high‐fat diet (HFD) in the form of Vanaspati ghee mixed in a 55% control diet and was given for 22 days to the groups G1, G2, G3, G4. The treatment plan is presented in Table 1.

**TABLE 1:** Grouping of rats and treatment plan.

Groups	Condition	Dosage
G0	Negative control	Normal diet
G1	Positive control	Disease group (HFD) but receive no treatment
G2	Porridge group 1	12 g (60% chickpea, 30% flaxseeds, 5% carrot chunks, 5% cinnamon)
G3	Porridge group 2	12 g (30% chickpea, 60% flaxseeds, 5% carrot chunks, 5% cinnamon)
G4	Porridge group 3	12 g (45% chickpea, 45% flaxseeds, 5% carrot chunks, 5% cinnamon)

### 2.5 Clinical Sign Observation

#### 2.5.1 Physical Parameter

Body weight measured from 0‐week first weight recorded, and second weight recorded at the end of trial 6‐week.

$$\begin{array}{c} \text{Weight gain}/\text{trial}:\text{Total weight gain}\;\left(g\right)\\ =\text{Final weight}\;\left(g\right)-\text{initial weight}\;\left(g\right) \end{array}$$

$$\begin{array}{c} \text{Weight gain}/\mathrm{day}:\text{weight gain}\;\left(g/\mathrm{day}\right)\\ =\text{Total weight gain}/\text{number of days}\;\mathrm{per}\;\text{trail} \end{array}$$

#### 2.5.2 Biochemical Parameters

For biochemical analysis, the rats were anesthetized with chloroform in an anesthesia chamber. As soon as the first signs of unconsciousness appeared, the rats were taken out, and heart punctures were used to collect blood samples, which were then placed in yellow serum‐separating vials. After that, the blood samples were centrifuged at 3700 rpm for 20 min at room temperature. After serum separation, the remaining serum was kept at −20°C to conduct further parameter examination (Albegali et al. 2021). A commercial kit from Merck Germany was used for the lipid profile, liver function tests (LFTs), and renal function tests (RFTs). A commercial kit (Merck, Germany) was used by enzymatic calorimeter for the lipid profile (TC, TG, LDL) using the formula LDL (Cholesterol) = TC − [TG/5 + HDL (Cholesterol)], HDL and very‐low density lipoprotein (vLDL) using the formula vLDL‐C (mg/dL) = TG/5 (Majeed et al. 2024). LFTs (alkaline phosphatase [ALP], aspartate aminotransferase [AST], alanine aminotransferase [ALT], total bilirubin [T‐bill]) were analyzed using a commercial colorimetric kit (Merck, Germany) (Hatipoglu et al. 2024). RFTs for creatinine and urea samples were analyzed using commercial kits (diagnostic kit from Merck, Germany) (Batool et al. 2024).

#### 2.5.3 Oxidative Stress Biomarkers Superoxide Dismutase (SOD)

A colorimetric test based on the suppression of nitro‐blue tetrazolium (NBT) reduction was used to detect SOD activity in order to evaluate oxidative stress in hyperlipidemia. The sera were centrifuged for 20 min at 5000 rpm and then cooled at 4°C. Following centrifugation, the obtained supernatant was kept for analysis at −20°C in Eppendorf tubes (Verma et al. 2022). 250 μL of 50 mM phosphate buffer (pH = 7.8) was mixed with 50 μL of NBT, 0.3 mM Ethylenediaminetetraacetic acid (EDTA), 100 μL of Triton X, 100 μL of methionine, and 400 μL of distilled water to measure the activity of SOD. The tubes were exposed to ultra‐violet (UV) light for 20 min after 50 μL of the supernatant and 50 μL of riboflavin were combined to form a reaction mixture. Lastly, sample absorbance was measured at 560 nm and contrasted with the reference (AlMasoud et al. 2024).

### 2.6 Histopathological Examination

Histopathological examination was done after slaughtering the experimental rats according to this method used by Karam et al. (2018). The liver, heart, and kidney tissues are examined histopathologically using a standardized process intended to maintain, process, and examine tissue morphology at the microscopic level. First, by biopsy, tiny representative samples usually about 1 cm^3^ in size are taken from each organ. To ensure ideal fixation, avoid autolysis, and preserve structural integrity, these tissues are submerged right away in 10% neutral buffered formalin. The specimens are examined grossly after fixation in order to find any obvious abnormalities or lesions and choose the best sections for additional processing. After that, the chosen tissues go through a number of processing stages, including xylene clearance, molten paraffin wax penetration, and dehydration using graded alcohols. To make sectioning easier, the tissues are fixed in paraffin blocks after they have completely penetrated. A microtome is used to cut thin sections, typically 3–5 μm thick, which are then placed onto glass slides. Hematoxylin and eosin (H&E) staining is commonly used on these slices to differentiate between cellular and extracellular components. E staining gives the cytoplasm and connective tissue a pink hue, but H staining tints nuclei blue. Lastly, a light microscope is used to assess histological characteristics, spot pathological changes, and help in diagnosis on the prepared slides.

### 2.7 Statistical Analysis

All analyses were conducted in triplicate, and results were expressed as mean ± standard deviation (SD). All pairwise comparison tests were conducted to check homogeneity among groups. Statistical differences among treatments were evaluated using one‐way ANOVA, followed by the completely randomized design (CRD) test. Data analysis was performed using software version 8.1 (Montgomery 2019).

## 3 Results and Discussion

### 3.1 Proximate Composition

The moisture content of the porridge samples ranged between 7.76% and 8.72%, with T1 showing the highest and T2 the lowest values. This variation may be attributed to differences in ingredient composition and water absorption capacity. The fat content showed significant differences among treatments, ranging from 16.15% to 26.16%, with the highest value observed in T2 due to its higher flaxseed proportion, as flaxseed is naturally rich in oil. In contrast, T1 exhibited the lowest fat content because of its lower flaxseed ratio, while T3 showed an intermediate value corresponding to its equal blend of chickpea and flaxseed. The protein content also differed significantly, ranging from 18.39% to 22.25%, with T3 showing the highest level, likely due to the balanced combination of both protein‐rich chickpea and flaxseed. T1 demonstrated a moderate protein level, whereas T2 had the lowest. The fiber content ranged from 14.83% to 18.16%, increasing with the flaxseed ratio, indicating that flaxseed contributed both soluble and insoluble fiber to the porridge. Thus, T2 had the highest fiber value, T1 the lowest, and T3 an intermediate level. The ash content, representing the total mineral composition, ranged between 1.85% and 2.82%, varying significantly across formulations. The highest ash content was found in T1, followed by T2, while T3 exhibited the lowest mineral concentration. Proximate composition of porridge across different treatments presents in Table 2. According to Chuwa and Dhiman (2021), ash content ranged between 1.7% and 2.36%, indicating the presence of essential minerals in small amounts. Protein content was relatively low (14.9%–18.2%), which may be due to the dilution effects of other components such as fat and carbohydrates or the denaturation of proteins during processing. Similarly, Haile and Shufa (2019) reported moisture content between 2.8% and 6.8%, reflecting good storage stability and reduced microbial activity. In another study, Jabeen et al. (2022) observed low fiber content (1.2%–10%), possibly due to processing losses, along with high fat content (23.9%–39.7%), attributed to the naturally rich lipid composition of flaxseed.

**TABLE 2:** Proximate composition of porridge across different treatments.

Treatment	Moisture	Fat	Protein	Fiber	Ash
T1	8.72 ± 0.17^a^	16.15 ± 0.14^c^	20.22 ± 0.32^b^	14.83 ± 0.12^c^	2.82 ± 0.09^a^
T2	7.76 ± 0.13^c^	26.16 ± 0.11^a^	18.39 ± 0.35^c^	18.16 ± 0.17^a^	2.35 ± 0.07^b^
T3	8.13 ± 0.19^b^	21.10 ± 0.17^b^	22.25 ± 0.39^a^	16.08 ± 0.14^b^	1.85 ± 0.05^c^

*Note:* T1 (2:1 chickpea‐dominant); T2 (1:2 flaxseed‐dominant); T3 (1:1 equal amount of both).

### 3.2 Body Weight

Body weight variations were monitored over a 45‐day period to evaluate the effect of chickpea‐ and flaxseed‐based porridge formulations on weight gain in HFD‐induced rats. The normal control group (G0), which received a standard diet without high‐fat induction, showed a moderate and steady increase in body weight, ranging between approximately 108 and 138 g representing normal physiological growth. In contrast, the hyperlipidemic control group (G1) fed a HFD without any treatment exhibited the highest weight gain, ranging from about 106 to 229 g confirming the obesogenic effect of the high‐fat diet through excessive lipid accumulation and disrupted metabolic regulation. Among the treatment groups (G2–G4), weight gain values ranged approximately from 74 to 98 g within moderate limits, remaining lower than the hyperlipidemic control but higher than the normal control. The T3‐based formulation (G4) demonstrated the least overall weight gain, followed by G2 and G3, indicating that porridge formulations, particularly the balanced chickpea and flaxseed blend, helped regulate body weight and counteract HFD‐induced obesity. The effect of porridge on weight is represented in Table 3.

**TABLE 3:** Effect of formulated porridge on physical parameter (weight) in HFD rats.

Groups	0 week (g)	6 week (g)	Total gain/trial (g)	Gain/day (g/day)
G0	108.64 ± 2.62^c^	138.45 ± 8.05^d^	29.81 ± 9.76^d^	0.66 ± 0.21^d^
G1	105.92 ± 2.52^cd^	228.65 ± 5.22^a^	122.73 ± 7.62^a^	2.73 ± 0.16^a^
G2	124.94 ± 4.54^a^	209.51 ± 8.42^b^	84.57 ± 12.4^bc^	1.88 ± 0.27^bc^
G3	100.79 ± 3.50^d^	199.06 ± 5.59^bc^	98.27 ± 8.63^b^	2.18 ± 0.18^b^
G4	116.50 ± 4.83^b^	190.75 ± 6.90^c^	74.25 ± 6.18^c^	1.65 ± 0.13^c^

*Note:* G0, negative control; G1, positive control (HFD group); G2, porridge group_1 (T1)_ 10 g (2:1); G3, porridge group_2 (T2)_ 10 g (1:2); G4, porridge group_3 (T3)_ 10 g (1:1). Values with different letters in the same column (a–d) are significantly different (*p* < 0.05) from each other.

The results of body weight gain per trial and per day showed a highly significant difference among groups 0.6–2.73 g as SD mentioned above (Ghasemi et al. 2021). This reduction in weight gain compared to G1 suggests a positive effect of chickpea‐dominant porridge, likely due to its high content of plant protein, resistant starch, and saponins, which help enhance satiety and inhibit fat accumulation. Although flaxseeds contain α‐linolenic acid and lignans with anti‐obesity potential, their higher fat content might have contributed to a relatively greater weight increase when not counterbalanced by sufficient fiber and protein. Collectively, these findings support that the formulated porridge especially the G4 blend may be effective in controlling diet‐induced weight gain and could serve as a therapeutic dietary approach for managing obesity and metabolic disorders This significant and maximum yet within normal average body weight gain occurred in clinically stressed rats by the HFD diet (Bravo et al. 2014).

### 3.3 Lipid Profile

The results revealed a significant difference (*p* < 0.05) in TC levels among the groups Table 4. TC values ranged between approximately 134 and 233 mg/dL. G1 exhibited the highest TC level, confirming the induction of hyperlipidemia through the HFD. In contrast, G0 maintained a significantly lower TC level (138.74 ± 2.57 mg/dL). Among the treatment groups, G4, which was given treatment 3 (T3, 1:1 porridge), showed the most pronounced reduction in TC (133.70 ± 2.61 mg/dL), which was statistically comparable to G0. G3, which received treatment 2 (T2, 1:2 porridge), demonstrated a notable decrease, followed by G2, treated with treatment 1 (T1, 2:1 porridge). All treatments (G2, G3, and G4) led to a significant (*p* < 0.05) reduction in TC levels compared to G1, with T3 (G4) being the most effective, suggesting its potential benefit for lipid profile improvement. In contrast, G0 had a significantly lower TG level (98.03 ± 2.25 mg/dL). A notable hypolipidemic effect was observed in all treated groups. G4, which received T3, showed the greatest reduction in TG, with the lowest level recorded at 88.64 ± 2.95 mg/dL. G2 and G3 also showed marked reductions compared to G1, demonstrating the lipid‐lowering potential of chickpea‐ and flaxseed‐based porridge. The lowest HDL level (24.49 ± 2.19 mg/dL) was observed in G1, indicating that the HFD had a detrimental effect on lipid metabolism. In contrast, G0 showed HDL levels within the desirable physiological range. Among the treated groups, G4 exhibited the highest HDL level, significantly higher than both the hyperlipidemic and normal control groups, demonstrating T3's strong potential to enhance “good cholesterol” concentrations. This improvement can be attributed to the synergistic effect of chickpea proteins and flaxseed‐derived omega‐3 fatty acids, which enhance reverse cholesterol transport and promote the synthesis of HDL. G3 also showed a considerable improvement, while G2 displayed a moderate increase compared to the hyperlipidemic control. Overall, HDL levels among the groups ranged approximately between 34 and 56 mg/dL, with all treated groups showing significant (*p* < 0.05) improvement over the HFD‐fed control. The LDL concentrations ranged from approximately 86–178 mg/dL and also varied significantly (*p* < 0.05) among the groups. G0 maintained much lower levels. Among the treated groups, G4 (T3) showed the greatest reduction in LDL, followed by G3 (T2) and G2 (T1). The highest reduction in G4 was due to the presence of soluble dietary fibers, especially β‐glucan and mucilage components from flaxseed, which bind bile acids and cholesterol in the intestine, promoting their excretion and reducing serum LDL levels. vLDL levels ranged between approximately 18 and 37 mg/dL, showing significant (*p* < 0.05) variation across groups. G1 exhibited the highest vLDL levels, confirming elevated triglyceride synthesis in response to the HFD. In contrast, G4 (T3) showed the lowest vLDL concentration, followed by G3 (T2) and G2 (T1). The marked reduction in vLDL among treated groups is likely due to the combined effects of omega‐3 fatty acids, plant sterols, and dietary fiber in flaxseed and chickpea, which enhance lipid metabolism by reducing hepatic triglyceride synthesis and improving lipoprotein clearance. Similarly, the results of the biochemical investigation also showed highly significant results that had a positive impact in improving biomarkers. The results of the lipid profile of TC, TG, LDL, and vLDL have shown maximum reduction and were highly significant among groups, but HDL has shown maximum increase among all groups, though all lipid parameters showed the best results in the G4 group. All lipid profile results were within the normal range.

During fermentation, fibers are converted into short‐chain fatty acids (SCFAs) such as acetate, propionate, and butyrate, which can modulate hepatic cholesterol synthesis and bile acid metabolism. Propionate, in particular, is known to inhibit HMG‐CoA reductase activity, thereby reducing cholesterol production. Moreover, soluble fibers can bind bile acids in the intestine, promoting their excretion and stimulating the liver to utilize circulating cholesterol for bile acid synthesis (Esmail et al. 2022). Whole flaxseed supplementation along with a HFD has been shown to significantly reduce TG, TC, LDL, and vLDL levels, as reported by (Srinivasa Naik et al. 2018). Similarly, (Dehiba et al. 2021) found that CPH and SPH both lowered TG, TC, LDL, and vLDLlevels and improved cardiovascular health, with CPH showing a stronger effect. These hydrolysates may serve as effective nutraceuticals or functional food ingredients. Another study (Elhassaneen and El‐Rahman 2020) also observed a significant TG, TC, LDL, and vLDL reduction with ginger root and flaxseed supplementation. Overall, these findings support the potential of plant and marine bioactives in managing hypertriglyceridemia. In contrast, (Mahmoud 2018), observed a more pronounced elevation in HDL levels in hypercholesterolemic rats supplemented with a mixture of flaxseed, sunflower, and pumpkin seeds. This combination demonstrated a strong modulatory effect, improving serum lipid levels and heart tissue conditions. The synergistic action of these seeds appears to enhance HDL concentration effectively, thereby decreasing the risk of CVDs. These findings support the inclusion of mixed plant seeds as functional dietary components for cardiovascular health management. Plant‐derived molecules are rich in bioactive compounds that can modulate lipid metabolism, decrease cholesterol absorption, and enhance the lipid profile (Sajid et al. 2025).

**TABLE 4:** Effect of formulated porridge on lipid profile (mg/dL) in HFD rats.

Groups	TC	TG	HDL	LDL	vLDL
G0	138.74 ± 2.57^c^	98.03 ± 2.25^d^	47.00 ± 3.33^b^	93.12 ± 3.22^d^	19.61 ± 0.45^d^
G1	232.81 ± 2.56^a^	183.52 ± 2.46^a^	24.49 ± 2.19^e^	178.37 ± 2.21^a^	36.71 ± 0.49^a^
G2	147.42 ± 2.64^b^	109.53 ± 3.28^b^	33.79 ± 3.46^d^	106.54 ± 2.18^b^	21.91 ± 0.66^b^
G3	142.22 ± 2.62^c^	102.04 ± 2.17^c^	40.62 ± 2.32^c^	101.48 ± 2.22^c^	20.41 ± 0.44^c^
G4	133.70 ± 2.61^d^	88.64 ± 2.95^e^	55.78 ± 2.62^a^	86.39 ± 2.57^e^	17.73 ± 0.59^e^

*Note:* G0, negative control; G1, positive control (HFD group); G2, porridge group_1 (T1)_ 10 g (2:1); G3, porridge group_2 (T2)_ 10 g (1:2); G4, porridge group_3 (T3)_ 10 g (1:1). Values with different letters in the same column (a–d) are significantly different (*p* < 0.05) from each other.

Abbreviations: HDL, high‐density lipoprotein; LDL, low‐density lipoprotein; TC, total cholesterol; TG, triglycerides; vLDL, very low‐density lipoprotein.

### 3.4 Liver Function Test (LFTs)

A notable increase in ALT activity was observed in the G1, indicating liver injury caused by the HFD. In contrast, the G0 showed ALT activity within the normal physiological range, reflecting healthy liver function. Among the treated groups, G4 exhibited the greatest reduction in ALT levels, followed by G3 and G2. The overall ALT activity among the groups ranged between approximately 25 and 65 u/L, showing a significant (*p* < 0.05) difference. The reduction in ALT levels among the treated groups suggests that the porridge formulations, particularly T3, helped restore hepatocellular integrity. This protective effect may be attributed to the antioxidant and anti‐inflammatory components of flaxseed (omega‐3 fatty acids, lignans) and chickpea (polyphenols and bioactive peptides), which prevent lipid peroxidation and reduce hepatic stress. AST levels ranged between approximately 46 and 89 u/L and varied significantly (*p* < 0.05) among the groups. In contrast, G0 showed much lower values, consistent with normal liver function. Among the treated groups, G4 exhibited the most notable reduction in AST, followed by G3 and G2, indicating that the porridge formulations effectively stabilized hepatic enzyme activity and reduced liver fat infiltration. The T‐bil concentrations showed a significant difference among groups (*p* < 0.05), ranging between approximately 0.5 and 1.3 mg/dL. The highest T‐bil level was recorded in the hyperlipidemic control, indicating impaired liver function and bile metabolism. Among the treatment groups, G4 showed the lowest T‐bil concentration, while G2 and G3 also exhibited reduced levels, reflecting improved hepatic clearance and reduced hemolytic stress. This decrease in bilirubin suggests that the bioactive components in the porridge enhanced liver detoxification efficiency. ALP activity ranged between 137 and 243 u/L, with significant differences (*p* < 0.05) among the groups. The G1 recorded the highest ALP levels, a marker of hepatic and biliary dysfunction. Conversely, G0 exhibited normal ALP activity, while the treated groups (G2–G4) showed gradual reductions, with G4 again recording the lowest values. The decline in ALP among treated rats indicates improvement in hepatic and biliary health. The effect of formulated porridge on LFTs is presented in Table 5. LFTs include ALT, AST, ALP and T‐bil showing a significant reduction among groups. All results of LFTs are within the normal range.

**TABLE 5:** Effect of formulated porridge on LFTs, RFTs, and SOD in HFD rats.

Groups	ALT (u/L)	AST (u/L)	T‐bill (mg/dL)	ALP (u/L)	Urea	Creatinine	SOD
G0	24.46 ± 3.85^d^	51.53 ± 2.97^d^	0.53 ± 0.34^b^	140.52 ± 2.60^cd^	25.36 ± 2.88^c^	1.06 ± 0.10^a^	2.35 ± 0.03^a^
G1	64.71 ± 4.63^a^	89.22 ± 2.86^a^	1.25 ± 0.13^a^	242.72 ± 4.79^a^	45.84 ± 2.08^a^	1.12 ± 0.16^a^	1.25 ± 0.02^d^
G2	39.38 ± 3.96^b^	74.72 ± 4.74^b^	0.65 ± 0.29^b^	154.57 ± 3.93^b^	36.56 ± 2.28^b^	1.02 ± 0.06^a^	2.07 ± 0.10^b^
G3	31.56 ± 2.17^c^	63.81 ± 4.10^c^	0.67 ± 0.22^b^	144.21 ± 3.53^c^	27.14 ± 2.33^c^	1.14 ± 0.21^a^	1.95 ± 0.13^c^
G4	28.66 ± 2.51^cd^	45.82 ± 5.08^d^	0.48 ± 0.17^b^	136.62 ± 3.63^d^	21.36 ± 1.96^d^	1.07 ± 0.17^a^	2.30 ± 0.06^a^

*Note:* G0, negative control; G1, positive control (HFD group); G2, porridge group_1 (T1)_ 10 g (2:1); G3, porridge group_2 (T2)_ 10 g (1:2); G4, porridge group_3 (T3)_ 10 g (1:1). Values with different letters in the same column (a–d) are significantly different (*p* < 0.05) from each other.

Abbreviations: ALP, alkaline phosphatase; ALT, alanine aminotransferase; AST, aspartate aminotransferase; T‐bill, total bilirubin.

In another study, we observed a more pronounced reduction in ALT, AST, and ALP levels in hypercholesterolemic rats treated with a mixture of flaxseed, sunflower, and pumpkin seeds. This seed combination contributed to improved liver enzyme profiles and hepatoprotective effects (Mahmoud 2018). A significant decrease in T‐bill levels was reported by Iahtisham‐Ul‐Haq et al. (2024), with values ranging from 0.94 to 0.81 mg/dL. The study found that the formulation with 12.5% incorporation of chickpea and mung bean exhibited a hypoglycemic effect along with improved liver function indicators. These findings suggest that the inclusion of legume‐based ingredients, such as chickpea and mung bean, in functional food products may contribute to better metabolic and hepatic health. A limitation of the study is the small sample size (*n* = 4 per group). This number aligns with previous studies conducted by Rabail et al. (2024), AlMasoud et al. (2024) in HFD‐induced metabolic changes (increased body weight, lipid profile alterations, etc.) where similar sample sizes (*n* = 5) effectively demonstrated significant biochemical and histopathological effects. No mortality was observed during the 45‐day study, and all rats were maintained under controlled conditions with minimal, safe dosages of the intervention, minimizing variability. While larger sample sizes could increase statistical power, managing a high number of animals presents practical challenges, including housing, handling, and maintaining controlled experimental conditions. Despite the limited sample size, consistent and reliable outcomes were observed. Future longer‐term studies could employ larger groups to further enhance statistical robustness.

### 3.5 Renal Function Test (RFTs)

A significant difference was found among the groups (*p* < 0.05). The highest urea level was observed in G1, indicating possible renal stress or dysfunction associated with lipid disorder. In contrast, G0 exhibited a lower urea level, reflecting normal renal function. Among the treatment groups, G4 showed the most notable reduction in urea, followed by G3, both of which were significantly lower than the HFD and comparable to or better than the normal group. G2 recorded a urea level of 36.56 ± 2.28 mg/dL, which was lower than G1 but higher than the other treatment groups. G1 and the treatment groups (G2, G3, and G4) all showed similar creatinine levels, around 1.00 mg/dL. G0 recorded a creatinine level of 1.06 ± 0.10 mg/dL. These findings suggest that renal filtration capacity was preserved across all groups, as neither the HFD nor the dietary treatments had a significant effect on creatinine. The effect of formulated porridge on RFTs is presented in Table 5. RFTs urea showed a significant reduction while creatinine showed a nonsignificant reduction.

A common indicator of renal function, urea is a crucial nitrogenous waste product produced in the liver and eliminated by the kidneys. Increased protein metabolism, dehydration, or compromised renal function can all be indicated by elevated blood urea levels. A significant decrease in urea levels was reported by (El Sayed and El Hawary 2019), indicating improved kidney function. A similar trend was observed with the supplementation of flaxseed, sunflower, and pumpkin seeds, where urea levels decreased from 41 to 37 mg/dL, suggesting the potential reno‐protective effect of this seed mixture. A waste product produced by muscle metabolism, creatinine is frequently employed as a kidney function indicator. Dietary intervention had minimal impact on kidney filtration function as reflected by this parameter. A similar trend was reported by (Rabail et al. 2024).

### 3.6 Oxidative Stress Biomarker Superoxide Dismutase (SOD)

The groups' differences were statistically significant (*p* < 0.05). Under normal physiological conditions, the G0 demonstrated the highest SOD activity (2.35 ± 0.03 mmol/dL), indicating excellent antioxidant defense. The HFD led to elevated oxidative stress, as reflected by the significantly lower SOD level in G1. The treatment groups showed clear improvements in SOD activity compared to G1, with G4, nearly restoring normal antioxidant status. G2 and G3 showed significant improvement in SOD levels. These findings indicate that all treatments significantly (*p* < 0.05) enhanced SOD levels compared to G1, with T3 (G4) providing the strongest antioxidant restoration, comparable to the G0. The effect of porridge on SOD is presented in Table 5. SOD is a vital antioxidant enzyme that converts superoxide radicals into hydrogen peroxide and oxygen, helping to safeguard cells against oxidative stress. A significant increase in SOD activity was reported by (Makni et al. 2011), indicating enhanced antioxidant defense. This effect was observed with the supplementation of a flaxseed and pumpkin seed mixture, suggesting their potential in boosting oxidative stress resistance and supporting overall cellular protection.

### 3.7 Histopathology of Vital Organs

#### 3.7.1 Heart

**FIGURE 1:**
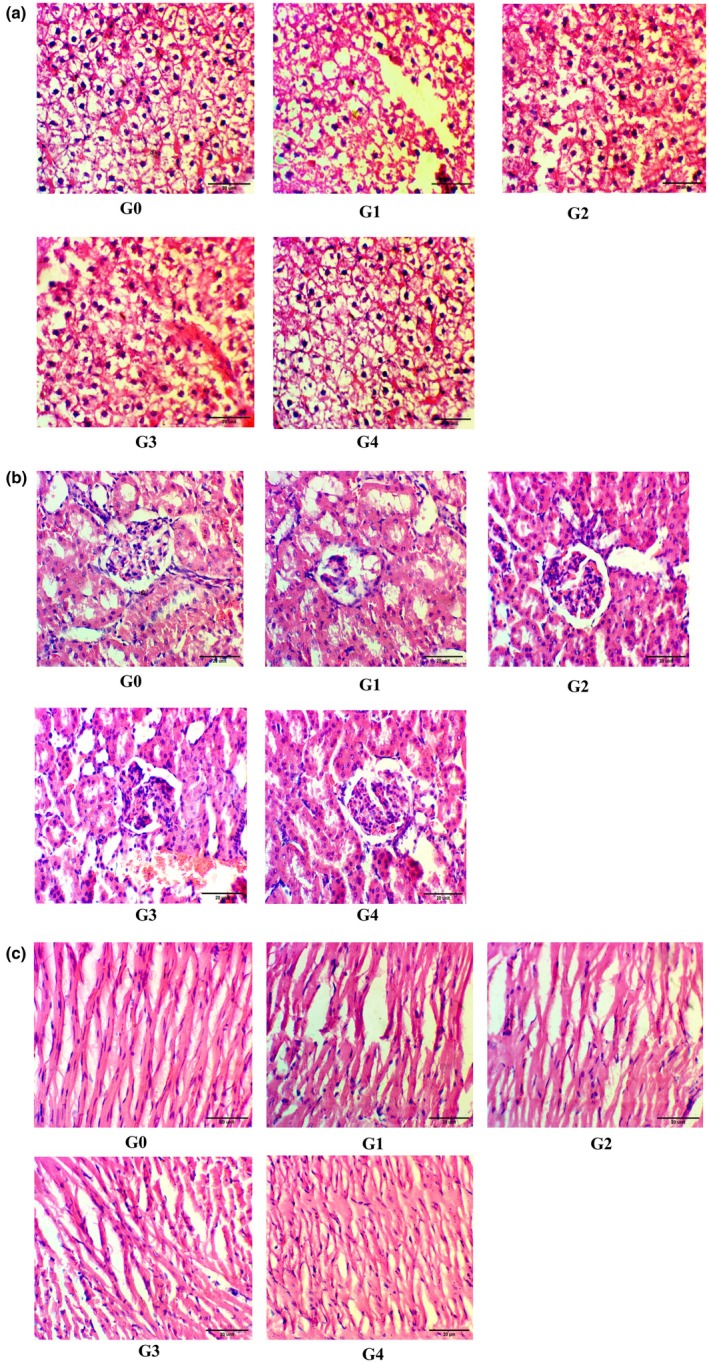
Histopathology of (a) heart, (b) kidney, and (c) liver.

G0 presented the normal pattern of the muscle fiber, mono‐nucleated myocytes, and well‐organized striations. G1 showed the disruption of the muscle fiber alignment, vacuolization lipid deposition in the myocardial fibers, interstitial edema, hypertrophy of the cardiac myocytes, fragmentation and loss of the striations In G2 and G3 mild restoration of the muscle fiber and striations, reduction in the fatty deposition, vacuolization and decrease in hypertrophy of myocytes were noticed. G4 revealed myocardial architecture towards normal, decreased hypertrophy, restored myocardial architecture, centrally located nuclei, uniform fiber alignment, organization of the muscle fibers. Histopathology of Heart shown in Figure 1a.

Supplementation of flaxseed showed a cardioprotective effect as reported by (Srinivasa Naik et al. 2018), which may be attributed to its high content of omega‐3 fatty acids, lignans, and dietary fiber that reduce lipid accumulation and oxidative stress in cardiac tissue. Histopathological findings also revealed improved heart architecture with reduced lipid deposition and restoration of normal myocardial structure, resembling the tissue of the normal group (Al‐Muslhi and Ali 2024).

#### 3.7.2 Kidney

G0 group showed normal glomerulus structure, nucleus located centrally and intact tubular structure. In G1 there was atrophy of the glomerulus, damage to renal tubular structure, interstitial edema, mesangial expansion, tubular vacuolization and nuclear pyknosis. In G2 and G3 groups, there was partial restoration of the glomerulus structure, improved tubular structure, reduction of vacuolization and necrosis. While in the G4 group, the glomerulus appeared to be normal, decreased interstitial edema, preserved tubular architecture, nuclei became intact and decreased mesangial expansion. Histopathology of the kidney is shown in Figure 1b. High‐fat diet consumption alters the normal renal function by modifying the cellular mitochondria. The renal changes compromise the ability of the kidney to recover from ischemia during reperfusion. The combined effects of dietary fiber, plant proteins, and anti‐inflammatory drugs that support renal structure and function under lipid stress show kidney histology as reported by (Prem and Kurian 2021).

#### 3.7.3 Liver

The histology of the liver demonstrated that in G0 the nucleus was uniform in the architecture, centrally located, the size of the hepatocytes was normal, no infiltration was seen. In G1 there were ballooning of the hepatocytes, steatosis, cytoplasmic vacuolization, congestion of the sinusoids, the nucleus became displaced towards the periphery, and there was a loss of uniform size and structure. In G2 and G3 they showed a mild reduction in steatosis, the nucleus was more central, and there was a decrease in vacuolization and size of hepatocytes. While the G4 showed the nucleus centrally located, architecture near to normal, a decreased pattern of infiltration, normalization of the sinusoidal structure. Histopathology of the liver is shown in Figure 1c. The dietary supplementation with crushed flaxseed is considered a potent antioxidant agent having a hepatoprotective role against steatohepatitis and fibrosis in an ovariectomized obese rat model as reported by (Sorour et al. 2022). Restoration of liver tissue and showed normal parenchyma and less fat accumulation as reported by (Saleem et al. 2024).

## 4 Conclusion

Hyperlipidemia, a group of disorders where elevated blood lipid levels are present, is common in wealthy nations and is the leading cause of coronary heart disease due to excess blood fat. Overall, all treatments show a significant reduction in all physical and biochemical parameters and improved histology of organs but T3 significantly enhances lipid profile, supports liver and kidney function, increases antioxidant activity, and preserves the histological structure of vital organs. This suggests a strong synergistic effect between chickpea and flaxseed, likely due to their complementary nutritional and functional components chickpea being rich in plant‐based protein, lysine, and potassium, while flaxseed contributes omega‐3 fatty acids, dietary fiber, lignans, and polyphenols. The presence of bioactive compounds in both chickpea and flaxseed not only aids in lowering blood lipid levels but also contributes to reducing oxidative stress and maintaining organ health. Chickpea–flaxseed‐based porridge shows excellent potential as a natural dietary approach to improve lipid metabolism and antioxidant status, supporting cardiovascular and metabolic health. Its development into convenient functional foods is encouraged, though human clinical trials are needed to confirm efficacy and safety.

## Author Contributions

**Sadia Zahid:** conceptualization, data curation, formal analysis, methodology, writing original draft. **Uswa Khawar:** formal analysis, methodology, writing review and editing. **Rehma Gulzar:** writing review and editing. **Usman Haider:** data curation, writing review and editing. **Ghalia Shamlan:** writing review and editing. **Isam A. Mohamed Ahmed:** writing review and editing. **Cristina Maria Maerescu:** conceptualization, data curation, writing review and editing. **Florin Leontin Criste:** writing review and editing. **Claudia Terezia Socol:** writing review and editing. **Rana Muhammad Aadil:** conceptualization, methodology, writing review and editing, resources, supervision.

## Ethics Statement

The animal study was approved by the Institutional Biosafety and Bioethics Committee (D#2275/ORIC) UAF, Pakistan. The study was conducted in accordance with the local legislation and institutional requirements.

## Data Availability

The original contributions presented in the study are included in the article; further inquiries can be directed to the corresponding author.
